# The effect of local application of tranexamic acid to reduce blood loss after off pump coronary artery bypass grafting (CABG)

**DOI:** 10.1186/1749-8090-10-S1-A131

**Published:** 2015-12-16

**Authors:** Mohammed Aslam Hossain, Shahriar Moinuddin

**Affiliations:** 1 Department of Cardiac Surgery, Bangabandhu Sheikh Mujib Medical University, Dhaka, 1000, Bangladesh

## Background/Introduction

After off-pump CABG surgery, bleeding and requirement of blood transfusion are the most common problems. Systemic use of antifibrinolytic reduces the postoperative blood loss. We have evaluated the effect of the topical use of tranexamic acid in the pericardial cavity on postoperative bleeding following open heart surgery.

## Aims/Objectives

To evaluate the effect of local application of tranexamic acid to reduce blood loss after off pump coronary artery bypass grafting (CABG).

## Method

Total 150 patients were enrolled in this double-blind, randomized, placebo-controlled, prospective clinical trial scheduled for primary isolated off pump coronary artery bypass grafting, divided in 2 groups (each group consisting of 100 patients). Patients with coagulopathies, renal failure, re-do surgery, or recent anti-platelet treatment were excluded. Tranexamic acid (TA) group (75 patients) received 1 gram of TA diluted in 100 ml normal saline. Placebo group (75 patients) received 100 ml of normal saline only. The solution was purred in the pericardial and mediastinal cavities before sternal closure. Postoperative blood loss, need for transfusion of blood products and the rate of re-sternotomy for bleeding were documented.

## Results

Both groups were comparable in their baseline demographic and surgical characteristics. In comparison with the placebo group, the patients receiving tranexamic acid had a significantly less chest tube drainage During the first 24 hours post-operatively up. In the tranexamic acid group (366 ± 158 mL) compared to the placebo group (580 ± 265 mL, p < 0.0001). There were no differences in mortality, morbidity between the 2 groups. More blood transfusions were administered to Placebo group patients (4.8 ± 1.61 units) as compared to Group I patients (2.46 ± 1.2 units, p < 0.0001). Re-exploration for excessive surgical bleeding in two patient in TA group, no difference was found in morbidity or mortality between both groups.

## Discussion/Conclusion

Topical application of tranexamic acid in patients undergoing primary coronary artery bypass grafting led to a significant reduction in postoperative mediastinal bleeding and requirement blood transfusion without adding extra risk to the patient.

